# Investigating ultra-high dose rate water radiolysis using the Geant4-DNA toolkit and a Geant4 model of the Oriatron eRT6 electron linac

**DOI:** 10.1038/s41598-024-76769-0

**Published:** 2024-11-04

**Authors:** Flore Chappuis, Hoang Ngoc Tran, Patrik Gonçalves Jorge, Sara A. Zein, Ioanna Kyriakou, Dimitris Emfietzoglou, Claude Bailat, François Bochud, Sébastien Incerti, Laurent Desorgher

**Affiliations:** 1 Institute of Radiation Physics (IRA), Lausanne University Hospital and University of Lausanne, Lausanne, CH-1007 Switzerland; 2 University of Bordeaux, CNRS, LP2I Bordeaux, UMR 5797, Gradignan, F-33170 France; 3 Medical Physics Laboratory, Department of Medicine, University of Ioannina, Ioannina, EL-45110 Greece

**Keywords:** UHDR simulation, FLASH effect, Geant4, Geant4-DNA, Chemical biology, Cancer, Computational science

## Abstract

Ultra-high dose rate FLASH radiotherapy, a promising cancer treatment approach, offers the potential to reduce healthy tissue damage during radiotherapy. As the mechanisms underlying this process remain unknown, several hypotheses have been proposed, including the altered production of radio-induced species under ultra-high dose rate (UHDR) conditions. This study explores realistic irradiation scenarios with various dose-per-pulse and investigates the role of pulse temporal structure. Using the Geant4 toolkit and its Geant4-DNA extension, we modeled the Oriatron eRT6 linac, a FLASH-validated electron beam, and conducted simulations covering four distinct dose-per-pulse scenarios – 0.17 Gy, 1 Gy, 5 Gy, and 10 Gy – all featuring a 1.8 µs pulse duration. Results show close agreement between simulated and experimental dose profiles in water, validating the eRT6 model for Geant4-DNA simulations. We observed important changes in the temporal evolution of certain species, such as the earlier fall in hydroxyl radicals ($^{ \bullet } \text{O}\text{H}$) and reduced production and lifetime of superoxide (${\text{O}}_{2}^{{\bullet\:}-}$) with higher dose-per-pulse levels. The pulse temporal structure did not influence the long-term evolution of species. Our findings encourage further investigation into different irradiation types, such as multi-pulse configurations, and emphasize the need to add components in water to account for relevant cellular processes.

## Introduction

In the constantly evolving field of radiotherapy, a groundbreaking approach has re-emerged less than ten years ago, holding the promise of reshaping the future of cancer treatment using ionizing radiation^1^. This approach lies on an enigmatic phenomenon – the FLASH effect – which materializes when high doses of radiation are delivered in a very limited time window, often less than a millisecond. This promising approach has attracted the attention of the scientific community due to its potential to spare healthy tissues while effectively targeting tumors. It represents a paradigm shift in radiotherapy, offering the prospect of fewer side effects, enhanced patient comfort, and improved treatment outcomes.

Despite these promises, the fundamental mechanisms driving the FLASH effect remain largely unknown^2,3^. To unlock the full potential of FLASH radiotherapy, it is imperative to gain a deeper understanding of the underlying mechanisms driving this effect and apply this knowledge to optimize treatment strategies^4^. Comprehending precisely how FLASH irradiation works demands a multidisciplinary approach, with Monte Carlo simulations holding a role. This represents an intricate challenge, requiring a comprehensive understanding of the impact of ionizing radiation on matter, with a particular focus on how radiation delivery modes influence the process.

Track structure Monte Carlo simulations of particle transport and their interactions with water span multiple orders of magnitude in time after irradiation but are typically limited to several microseconds^5–7^. These simulation toolkits are designed for accurate modeling of the early radiation effects on water. In this framework, fundamental questions arise for ultra-high dose rate (UHDR) irradiation relevant to FLASH radiotherapy^8^: to what extent must we delve into the details of the irradiation structure to model a FLASH irradiation? Do we require precise simulations right from the start of irradiation, or could it be that detailed modeling of early temporal events holds minimal significance for the longer-term outcomes? These questions form the basis of our study, guiding an exploration of ultra-high dose rate irradiation, as well as our efforts to establish potential connections between early radiation effects and the biological FLASH effect.

This study goes beyond simulations, integrating them as far as possible with experiments. Our objective is to simulate water radiolysis^9^ under ultra-high dose rate conditions, with the use of a real irradiation source from an existing FLASH-validated modality. While traditional approaches rely on generic particle sources with predefined energy and propagation directions^10–13^, we adopt a distinctive approach by using the general-purpose Geant4 toolkit^14–16^ to model the Oriatron eRT6 linac prototype – an electron beam modality dedicated for FLASH effect studies^17^. The eRT6 irradiation device has been widely used in various research studies related to FLASH radiotherapy^18–23^. Geant4 is a polyvalent particle transport simulation toolkit. Through this modeling, we aim to faithfully replicate the precise conditions of irradiation encountered in typical experimental scenarios. This includes capturing critical factors such as energy spectra, directional dependencies, and temporal structures.

Validated with experimental data, this eRT6 model then becomes the basis for exploring the impact of dose-per-pulse and irradiation temporal structures on the production of radio-induced species in water using Geant4-DNA^5,24–26^. In addition, the timescales involved with Geant4-DNA simulations necessitate an additional strategy. Geant4-DNA becomes increasingly time-consuming when exploring processes occurring beyond microseconds. To bridge this gap, we employed a differential equation solver to extend the temporal scope of water radiolysis simulations toward homogeneous chemical processes, aligning them with experimental observations that often span durations far exceeding microseconds^19^.

## Materials and methods

### Geant4 model of the Oriatron eRT6 linac and its validation

**Fig. 1:**
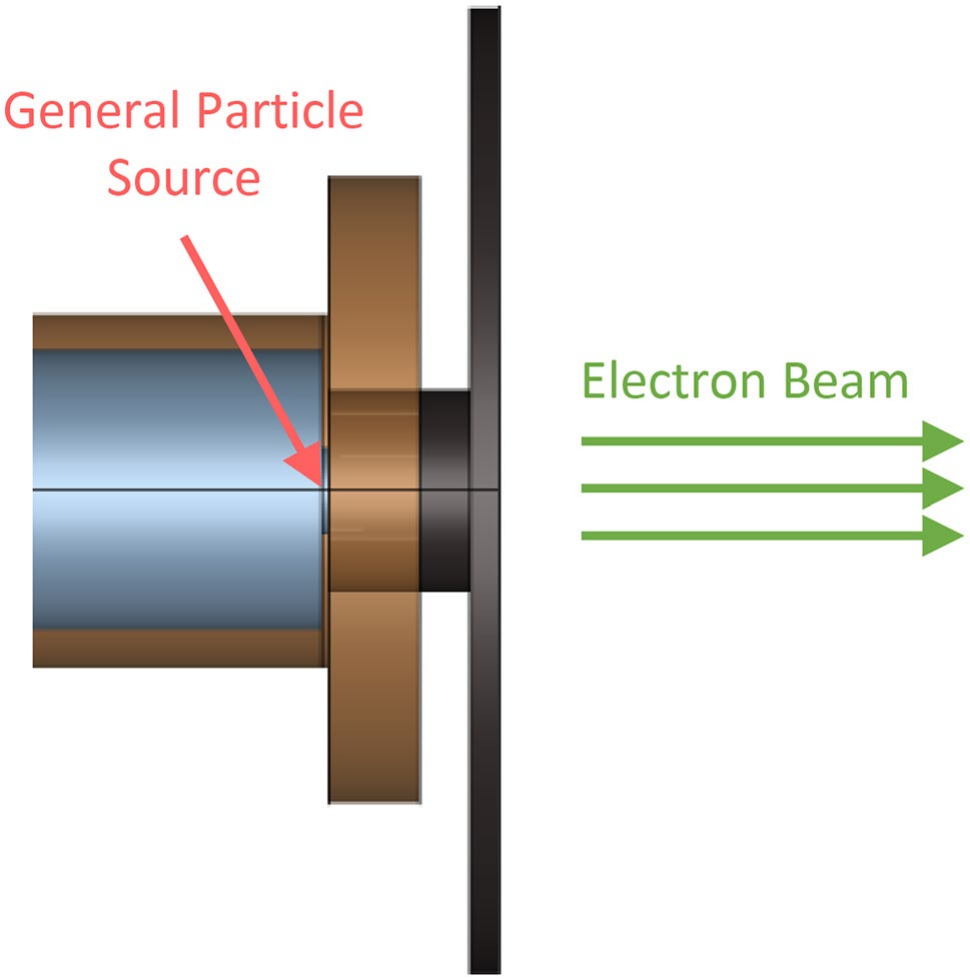
Head of the Oriatron eRT6 linac as modeled in Geant4. The figure additionally indicates the location of the primary electron source, generated by the Geant4 General Particle Source module. Materials of the visible elements: copper (brown) and carbon (black). Light blue represents the vacuum.

Modeling the eRT6 beam using the Geant4 toolkit (release 11.1) involves two distinct aspects: defining the linac geometry and generating its particle source. Figure 1 provides a visual representation of the former, specifically the head of the eRT6. This geometric configuration comprises a series of nested elements. Notably, it incorporates an exit window constructed from nickel (approximately located at the position indicated by the red arrow), followed by a succession of hollow cylinders, some of which are constructed from carbon for collimation purposes. Inside this geometric structure and immediately prior to the exit window, we generate the primary particle source in vacuum, consisting of the electrons at the end of the accelerating cavity. As a result, the simulation begins at the positions where electrons are generated and proceeds toward the right, reaching the far end of the eRT6 head. From there, it becomes possible to introduce any desired components in front of the beam.

To validate the eRT6 modeling, we computed 3D dose maps in a 30$\:\times\:$30$\:\times\:$30 cm^3^ water tank to obtain beam profiles and a depth dose curve. These results are compared with experimental dose profiles acquired under identical geometrical conditions at the eRT6 facility. Geant4 includes a user-built feature for straightforward dose computations in water using a 3D cartesian scorer, essentially a mesh designed for dose scoring. The water tank is positioned at various distances from the eRT6, specifically at 34 cm, 46 cm, and 95 cm. These distances are typically referred to as SSD for Surface to Source Distance, and each corresponds to a standard irradiation scenario in UHDR mode: 10 Gy/pulse for SSD = 34 cm, 5 Gy/pulse for SSD = 46 cm, and 1 Gy/pulse for SSD = 95 cm.

Source generation is performed using the Geant4 General Particle Source (GPS) tool, enabling characterization of the spectral, spatial, and angular distributions of the particle source^27^. For modeling the eRT6 source, we used a “beam” source position distribution, with a circular plane shape featuring a 1 mm beam spot (representing the radial standard deviation of the beam position profile). Particle energy is a critical parameter; lacking a precise and reliable energy spectrum for the eRT6, we decided to treat it as a tunable parameter. We started with an estimated spectrum provided by the constructor and fine-tuned it to achieve the best possible agreement with the experimentally obtained dose profiles. To conclude on the simulation approach, all computations employed the Geant4 built-in Shielding Livermore physics list.

The acquisition of experimental profiles at the eRT6 facility included relative measurements of Percentage Depth Dose (PDD) and lateral dose profiles. These measurements were conducted using a solid water phantom constructed from RW3 slabs (PTW-Freiburg GmbH, Freiburg, Germany) and EBT3 Gafchromic films (Ashland Specialty Ingredients G.P., Bridgewater, NJ, USA). Further details regarding dosimetry procedures can be found in existing literature^17,28^.

To measure the PDD, we performed a series of four distinct irradiations. During each irradiation, three or four films were placed between solid water slabs, each slab having a thickness of 10 mm. The first film was positioned at various initial depths (0, 2, 5, or 7 mm), and measurements were conducted up to a depth of 35 mm. The SSD was 34 cm and the dose was delivered in a single pulse lasting 1.8 µs, equivalent to 10 Gy. Lateral dose profiles were measured at a depth of 12 mm for the three SSD values described above: 34 cm, 46 cm, and 95 cm. This choice of SSD distances resulted in the delivery of 1, 2, and 10 pulses respectively to accumulate a total dose of approximately 10 Gy on the film. Each pulse had a duration of 1.8 µs, and the pulse repetition frequency was maintained at 100 Hz.

### Approach used for studying ultra-high dose rate irradiation

**Fig. 2:**
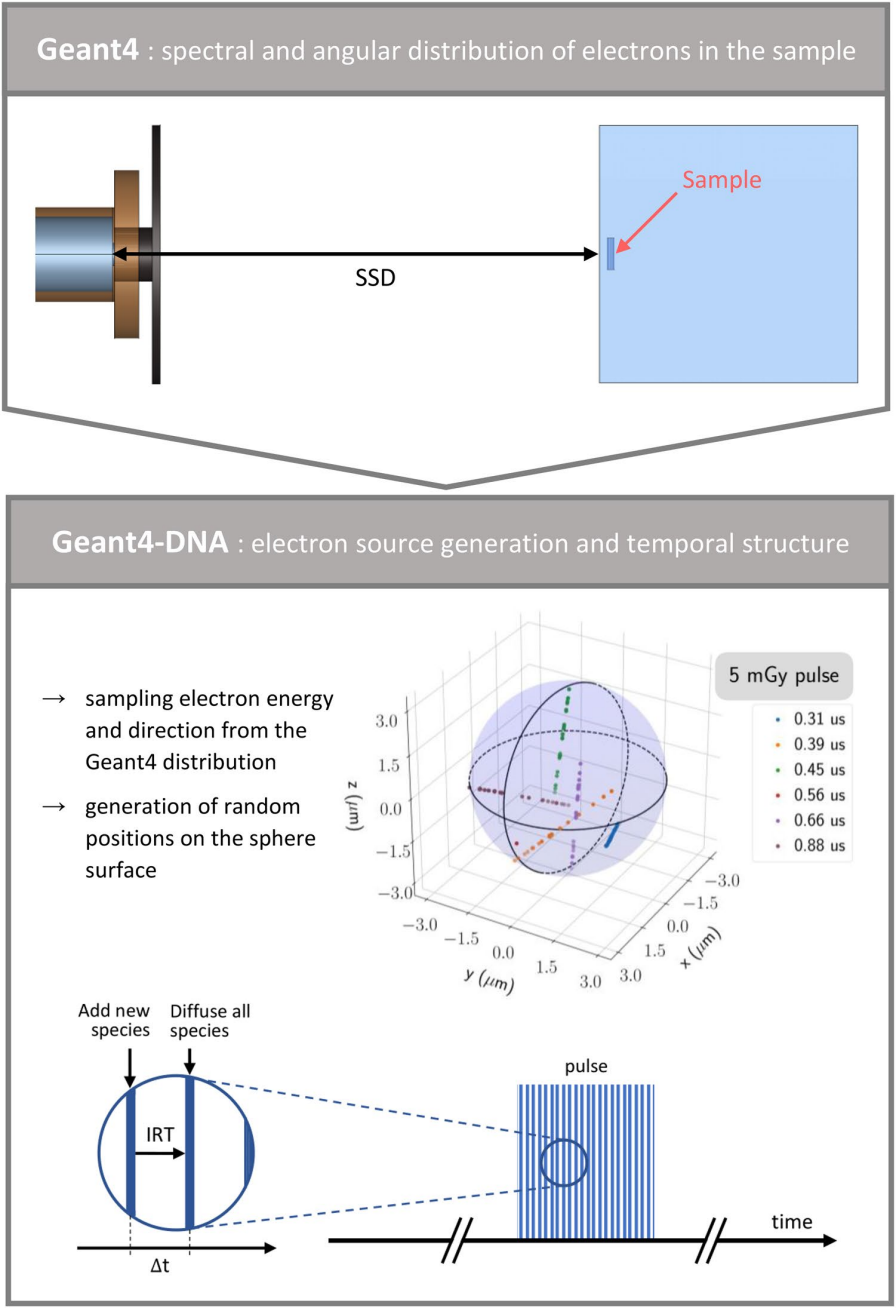
Approach used for studying ultra-high dose rate irradiation using the general-purpose Geant4 toolkit and its Geant4-DNA extension. Geant4: modeling of the irradiation facility in full-scale conditions, including the eRT6 linac and the irradiated sample housed within the water tank. The sample acts as a computational volume for characterizing electron distributions. Geant4-DNA: geometry of the simulated water system, with the sphere surface serving as a source for the eRT6-specific electrons. For illustrative purposes, the colored dots indicate the positions of excited/ionized water molecules and solvated electrons arising from various primary electrons during a 1 µs pulse of 5 mGy. The emission time points associated with each primary electron are also displayed, illustrating the temporal structure of the irradiation. Δt denotes the time interval between successive electrons or bunches of electrons within the simulated pulse.

The approach outlined in Fig. 2 firstly relies on modeling a FLASH-validated beam, in this case, the eRT6 linac. This beam model serves as the fundamental basis for simulating the standard radiochemical experiments performed at the eRT6. In these experiments, a sample is placed inside a water tank, precisely 13 mm from its entrance^19^. The tank is positioned at varying distances (SSD) from the linac, with each SSD corresponding to a specific dose-per-pulse scenario. The sample itself is the focal point for studying the water radiolysis process. For these experiments, it is an Eppendorf tube, which we chose to represent within the simulations as a cylinder measuring 8.8 mm in diameter and 37 mm in height (approximately a 2 ml Eppendorf tube). The key objective is to accurately determine the effective irradiation of the sample. By harnessing the capabilities of Geant4 and the geometric setup depicted in Fig. 2, we simulated the eRT6 beam and collected irradiation details within the sample. This data set encompasses both the spectral and angular distributions of electrons as they traverse the sample, and is used to determine the spectral and angular distributions of the average electron fluence. Notably, as electron energy is intrinsically correlated with its direction in this irradiation geometry, we effectively computed a joint distribution of both electron energy and direction.

This Geant4 simulation corresponds to the initial step of the approach: simulating the eRT6 beam and gathering data on the joint distribution of electron energy and direction within the sample. The subsequent phase is to leverage this distribution to create a source tailored for a Geant4-DNA water radiolysis simulation, but also specifically designed to replicate the typical irradiation conditions of the eRT6 (Fig. 2). Running water radiolysis simulations at the sample scale using Geant4-DNA is not yet achievable, given the current computational constraints. Therefore, we adopted a spherical geometry to simulate the electrons generated by the source, that is, the physical interactions between electrons and water molecules are limited within a 2 μm radius sphere. This radius is large enough to guarantee an adequate number of physical interactions, but not so large as to produce an excessive amount of radio-induced species, which would be resource intensive. The spatial restriction does not apply to chemical interactions among radio-induced species, which can occur both within and beyond the defined sphere. Indeed, the overall water volume is dimensioned to be sufficiently large, ensuring that no species reach the system boundaries. The electron source is generated on the sphere surface, and its characteristics are derived from the previous Geant4 simulation. Specifically, the spectral and angular distribution of electrons computed using Geant4 serve as the basis for defining the source parameters in Geant4-DNA. In particular, the electron source is generated using the following steps:

A. Sampling the energy and direction of electrons from the Geant4 joint distribution,
B. Sampling the initial position of each electron on the sphere surface, following this methodology:

- Step n°1: randomly selecting a position (*x*) on a 2 μm radius disk,
- Step n°2: rotating the system based on the previously sampled direction from stage A and calculating the new coordinates of point *x*,
- Step n°3: defining the line that passes through point *x* and follows the direction sampled in stage A,
- Step n°4: identifying the two intersection points where the line intersects with the sphere surface and selecting the one allowing the electron to propagate toward the center of the sphere.

In simpler terms, we randomly select a position on the section of the sphere perpendicular to the electron propagation direction established in stage A, and we then determine the correct intersection point between the line defined by both this random position and the electron direction, and the sphere surface.

This process makes it possible to characterize the electron source for modeling ultra-high dose rate irradiation. During the Geant4-DNA simulation, electrons are generated one by one until their physical interactions collectively reach a pre-defined dose threshold within the sphere, effectively defining a single pulse. Conversely, the total cumulated dose inside the sphere defines the number of electrons simulated to form a pulse. To introduce a temporal structure, a random time point is assigned to each primary electron emission within the pulse according to a user-defined distribution. Several electrons can share the same emission time point to speed up the simulation, thus forming bunches of electrons within the pulse. Radio-induced species produced by these electrons are added into the system at the specific times associated with their respective electrons. Given our use of the independent reaction times (IRT) method^29^, we diffuse species already present in the system upon the arrival of the new species, enabling the computation of an updated IRT chemical reactions table. Users retain the flexibility to select the pulse structure and the dose-per-pulse.

### Extended physics models for Geant4-DNA

The Geant4-DNA toolkit (release 11.1) currently has physics models for electron inelastic interactions with liquid water, covering an energy range from a few eV to 1 MeV using the default “DNA_option 2” physics constructor^25^. Additionally, users have the option to select alternative physics constructors: “DNA_option 4”, which spans an energy range from 10 eV to 10 keV, and “DNA_option 6”, covering the energy range from 11 eV to 256 keV^30,31^. Given that the nominal energy of the eRT6 beam lies between 5 MeV and 6 MeV, the limits of the existing physics models are insufficient to accurately simulate ultra-high dose rate irradiation scenarios with this facility. Consequently, an extension was deemed necessary to ensure precise simulations across the entire spectrum of electron energies.

The “DNA_option 4” constructor implements electron excitation and ionization based on cross sections derived from the dielectric theory of inelastic scattering. In a recent study, these inelastic cross sections were extended up to 1 MeV, incorporating relativistic corrections and an improved dielectric response function^32^. Subsequently, this model was further extended up to 10 MeV. We exclusively employed this extended version of the “DNA_option 4” constructor to obtain the results presented in this study.

### Temporal extension of water radiolysis simulations

To extend the temporal scope of Geant4-DNA water radiolysis simulations well beyond several microseconds, we adopted an approach designed for simulating homogeneous chemical processes. The objective is to transition directly from the Geant4-DNA Monte Carlo methodology, which accounts for system inhomogeneity, to a homogeneous modeling paradigm using a differential equation solver. The selected solver relies on Python’s built-in packages and was validated using the CHEMSIMUL software, renowned for its proficiency in simulating chemical kinetics, notably radiolytic processes involving pulse trains^33^. This approach centers on solving a system of differential equations, defined by the list of chemical reactions considered for the investigation and their respective rate constants. We used the “integrate” package included within the SciPy Python module, and in particular the “LSODA” method provided by the “solve_ivp” function of this package^34^. This function enables the numerical integration of a system of ordinary differential equations when provided with initial values.

### Investigated irradiation scenarios and quantification of radiolytic species production

From modeling the Oriatron eRT6 linac to our approach for studying ultra-high dose rate irradiation and extending the temporal scope of simulations, we combined these individual components to simulate single pulses across various irradiation scenarios, using Geant4 and Geant4-DNA (release 11.1). Table 1 presents a comprehensive list of parameters for these scenarios. A first set of conditions explores different dose-per-pulse values, including 0.17 Gy, 1 Gy, 5 Gy, and 10 Gy. A second investigation aims to study the significance of the irradiation temporal structure by comparing an instantaneous 5 Gy pulse with its temporally modulated counterpart. For all simulations, we used a 2 μm sphere radius, performed the Geant4-DNA computations up to 5 µs post-irradiation, and included 1% oxygen in water at neutral pH: [$\:{\text{H}}_{3}{\text{O}}^{+}$] = [$\:{\text{O}\text{H}}^{-}$] = 1.0$\:\cdot\:$10^-7^ M at 25 °C^35^. The pulse temporal structure has a rectangular shape in all irradiation scenarios. The list of chemical reactions is available in Table S1 of the Supporting Materials.

**Table 1:** Summary of parameters for the different irradiation scenarios presented in this study.

Dose (Gy)	Pulse width (µs)	# electrons per bunch	SSD (cm)
0.17	1.8	1	190
1	1.8	10	95
5	0 ^a^	–	46
5	1.8	50	46
10	1.8	500	34

^a^ instantaneous pulse (all electrons in the pulse have an identical emission time).

The simulation outcomes are the amount of radiolytic species produced in the irradiated system. These results are expressed using the *G* value parameter, which is defined as “the mean number of entities produced, destroyed, or changed by an energy imparted of 100 eV”^36^. In all the reported results, we determined the *G* values based on the total energy imparted. In other words, *G* values computed during irradiation are derived from the energy imparted by the entire pulse, rather than the energy imparted up to the specific moment being considered within the pulse.

## Results

### Geant4 model of the Oriatron eRT6 linac and its validation

**Fig. 3:**
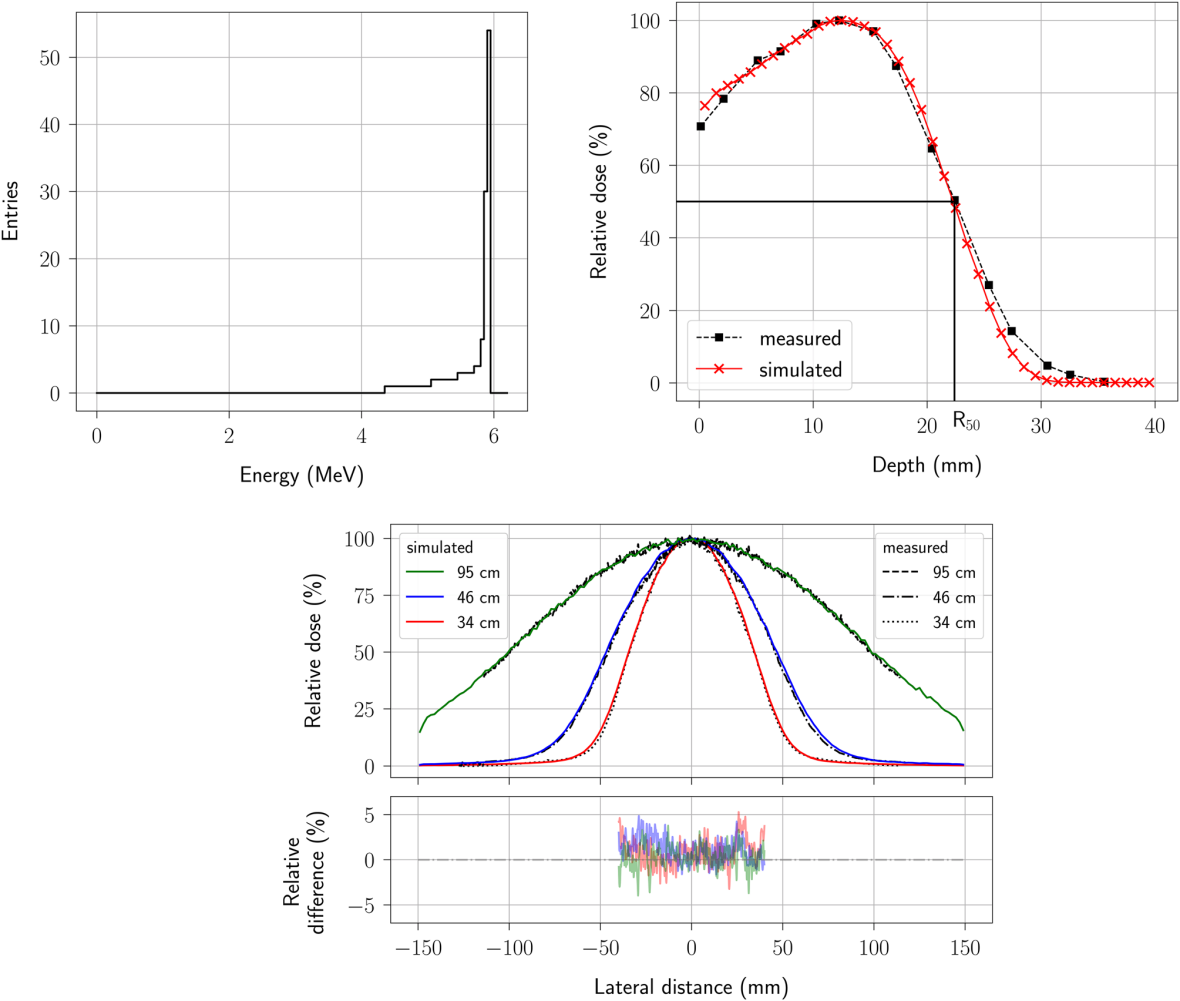
Energy spectrum of the primary electron source, a depth dose curve and beam profiles of the Oriatron eRT6 linac. Top left: optimized energy spectrum of the eRT6 with respect to the simulated dose profiles. Top right: percentage depth dose (PDD) in water for the SSD = 34 cm scenario. Bottom: lateral dose profiles at a 12 mm water depth for the SSD = 34 cm, SSD = 46 cm, and SSD = 95 cm scenarios.

The design of the simulated eRT6 beam includes two tunable parameters: the spectral distribution of electrons before reaching the linac exit window and the beam spot, which we set at 1 mm. Figure 3 showcases the spectrum selected after the optimization process, alongside the corresponding dose profiles in water and their measured counterparts. The PDD curve notably highlights the R_50_ value^37,38^. Figure 3 also displays the relative difference between the simulated and measured lateral dose profiles for the different chosen SSD distances.

### Generating the electron source for Geant4-DNA water radiolysis simulations

**Fig. 4:**
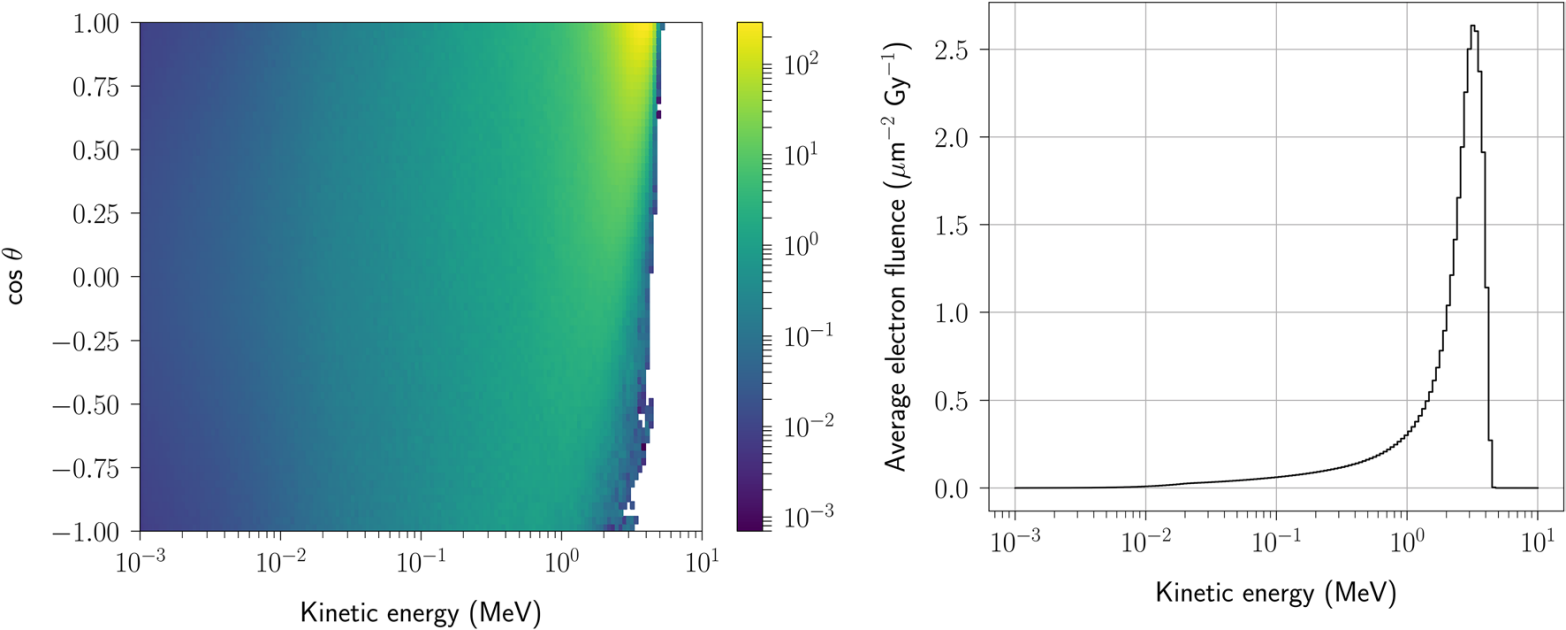
Spectral and angular distribution of electrons within the sample irradiated by the eRT6 beam. Left: joint distribution of electron energy and direction, used to define the Geant4-DNA electron source. θ is the polar angle of the spherical coordinate system. Right: energy spectrum of all electrons traversing the irradiated sample. The SSD distance parameter equals 46 cm, which corresponds to the 5 Gy/pulse scenario.

**Fig. 5:**
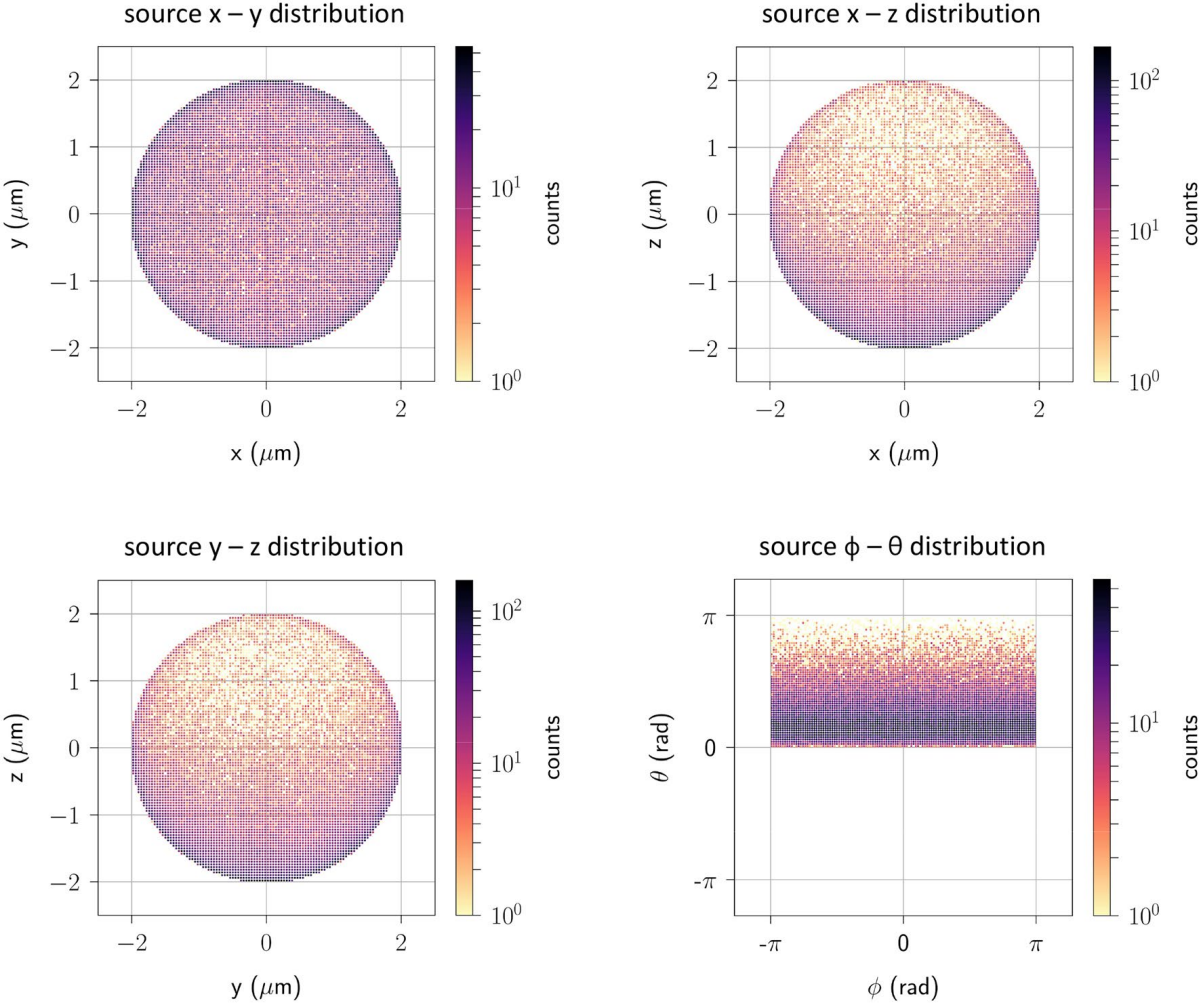
Spatial and angular distributions of the electron source for the Geant4-DNA ultra-high dose rate simulations. The SSD distance parameter equals 46 cm, which corresponds to the 5 Gy/pulse scenario. θ and ϕ are the polar and azimuthal angles of the spherical coordinate system.

Figure 4 shows the distributions characterizing electrons passing through the irradiated sample during the simulation of the eRT6 beamline and its experimental configuration (Fig. 2). The electron beam propagates along the z-axis of a Cartesian coordinate system. The figure on the left presents the joint distribution representing the relationship between electron energy and direction, while the figure on the right only displays the energy spectrum of electrons interacting with the water molecules of the sample. The joint spectral and angular distribution forms one component of the electron source customized for Geant4-DNA simulations. The second aspect of the approach involves defining the spatial distribution of this source. Figure 5 illustrates the occurrence of positions where primary electrons from the source are generated. Similar to Fig. 4, this illustration also gives the direction of the generated primary electrons.

### Investigated irradiation scenarios and quantification of radiolytic species production

**Fig. 6:**
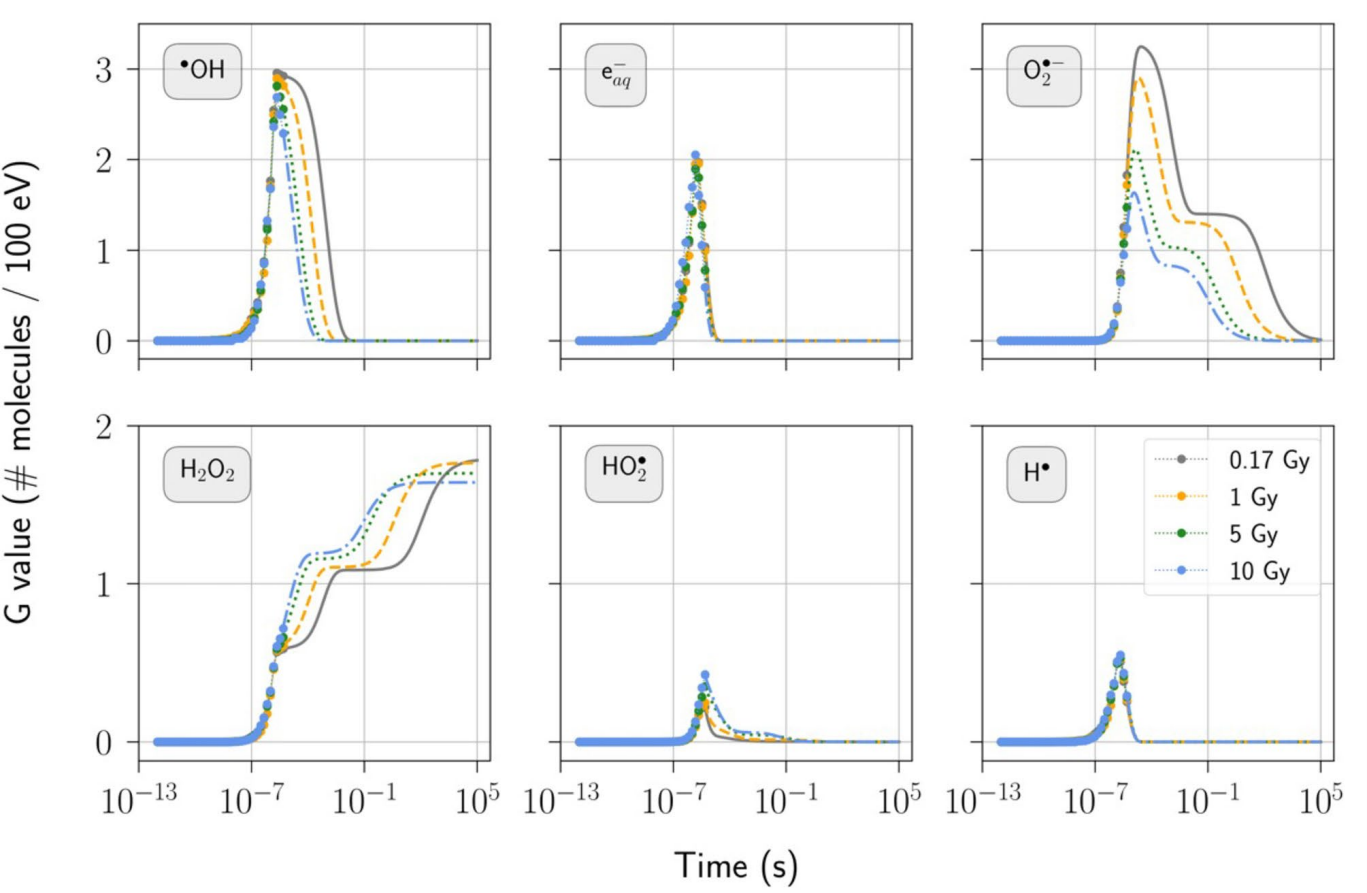
Temporal evolution of selected radio-induced species following single pulses of 0.17, 1, 5, or 10 Gy, each with a pulse duration of 1.8 µs. The number of electrons per bunch varies with dose: 1, 10, 50, and 500, respectively. The water medium contains 1% oxygen and has a neutral pH. Geant4-DNA ran from time 0 to 5 µs, at which point the differential equation solver took over to simulate the homogeneous phase. The shift in marker style indicates the transition between these two methods. *G* values given during a pulse were computed using the energy imparted by the entire pulse.

**Fig. 7:**
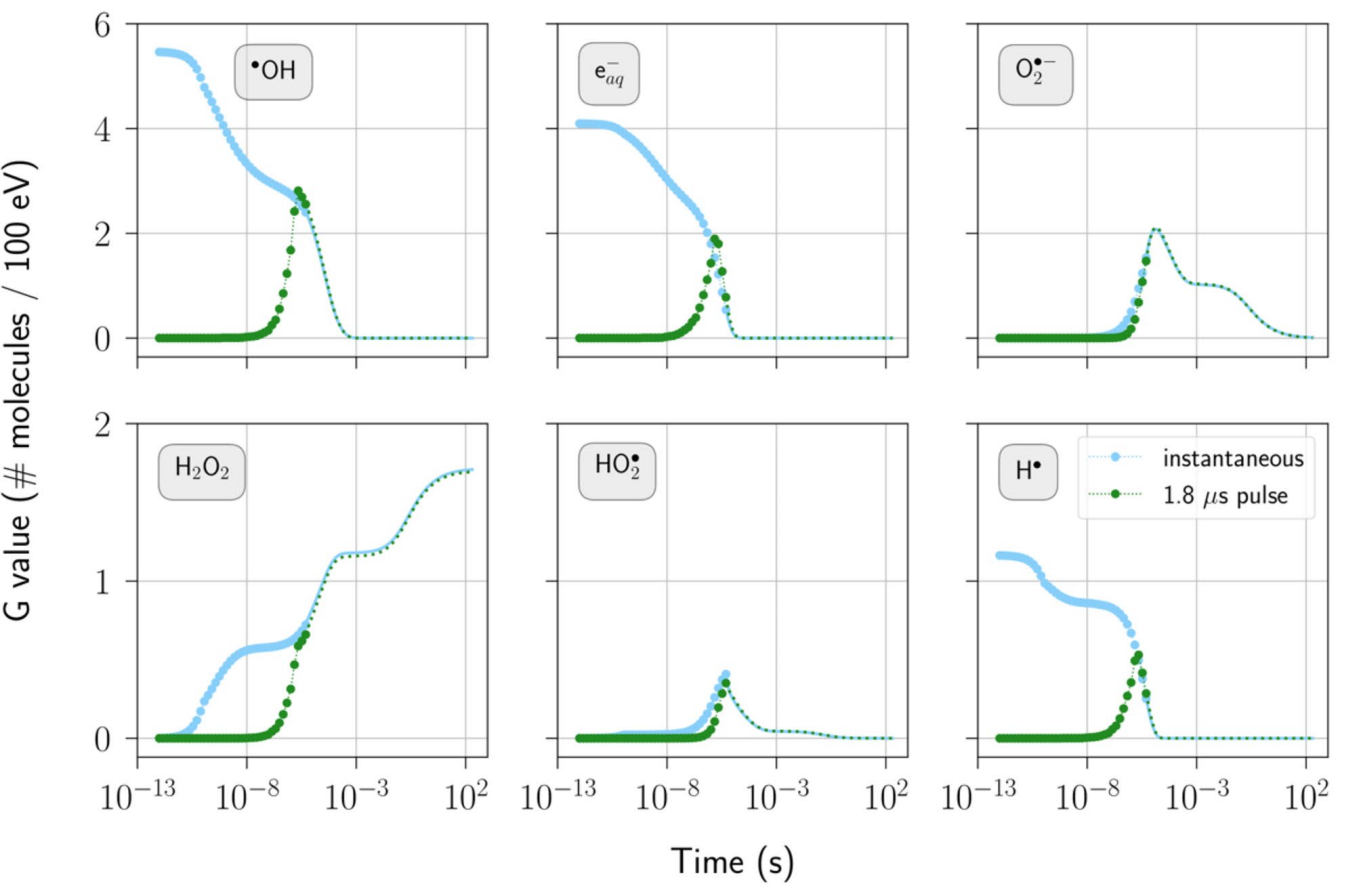
Temporal evolution of selected radio-induced species following a single 5 Gy pulse under two irradiation scenarios: one with a 1.8 µs pulse duration as well as 50 electrons per bunch, and the other being instantaneous. The water medium contains 1% oxygen and has a neutral pH. Geant4-DNA ran from time 0 to 5 µs, at which point the differential equation solver took over to simulate the homogeneous phase. The shift in marker style indicates the transition between these two methods. *G* values given during a pulse were computed using the energy imparted by the entire pulse.

Figure 6 presents the temporal evolution of selected radio-induced species obtained with the electron source tailored to replicate irradiation by the eRT6 linac. This includes four practical irradiation scenarios involving single pulses, each with varying dose-per-pulse levels ranging from 0.17 Gy to 10 Gy. In all cases, the pulse duration remains constant at 1.8 µs. Figure 7 illustrates the scenario where the 5 Gy pulse is delivered instantaneously, contrasting it to its equivalent featuring a temporal structure. Both figures merge Geant4-DNA Monte Carlo simulation results with the temporal extension outcomes achieved through the differential equation solver, providing comprehensive coverage of the entire temporal evolution of the chemical system.

## Discussion

### Geant4 model of the Oriatron eRT6 linac and its validation

The Geant4-based design of the eRT6 linac demonstrates its effectiveness given the satisfactory alignment of simulated dose profiles and experimental data (Fig. 3). The careful calibration of the tunable parameters based on extensive optimization using these experimental data allowed for the model to accurately represent the actual eRT6 facility. Such alignment with experimental outcomes ensures that the model is well adapted and serves as a validation of our modeling approach.

More specifically, after the optimization process, the energy distribution of eRT6 electrons is characterized by a spectrum featuring a peak between 5 MeV and 6 MeV. The beam energy is key for shaping the PDD profile and therefore the eRT6 spectrum influences the PDD obtained. In our optimization efforts, we focused primarily on key regions of the PDD, specifically those of utmost importance for experimental applications, that is, around the maximum relative dose. Consequently, we attributed lesser significance to the tail dose components, as this aspect holds relatively lower experimental relevance. As a result of the optimization process, the calculated R_50_ value closely aligns with the measured value, with R_50_ = 22 mm. This parameter is a common metric for reference dosimetry of therapy electron beams^37,38^. The concordance between calculated and measured R_50_ values underscores the overall accuracy and effectiveness of the model. The PDD profile generated through our simulation exhibits commendable agreement with experimental data, affirming in this situation the reliability of our modeling approach.

Concerning the lateral dose profiles, an important determinant of their shapes lies in the beam spot, which we selected to be 1 mm diameter. This critical factor contributes to the beam angular dispersion. With the tuned parameters of the eRT6 model, the simulation results closely mirror the experimental data, exhibiting a relative difference within a narrow margin of 5% in the central region of the profiles. This central area, representing the focal point of irradiation, holds the most significance in practical applications since sample or other animal models are placed at this location. The alignment between our simulations and experimental observations within this critical region underscores the precision of the model in this case.

### Generating the electron source for Geant4-DNA water radiolysis simulations

The joint distribution of electron energy and direction, presented in Fig. 4, provides a clear illustration of the expected correlation between energy and direction of electrons within the simulated sample. In particular, electrons with the highest energies predominantly align with the initial beam direction, an observation in line with expectations. This alignment is a consequence of the reduced scattering experienced by high-energy electrons, making them more likely to maintain their trajectory^39^. In contrast, as electrons undergo multiple physical interactions, particularly when they lose a significant amount of energy, they become increasingly prone to scattering, resulting in a less predictable path. Additionally, the generation of secondary electrons during these interactions introduces lower energy electrons into the system. While the direction of these secondary electrons initially depends on the incident particle direction, their inherently lower energy levels allow them to disperse rapidly in various directions. Consequently, low energy electrons within the sample have an isotropic propagation pattern, lacking a preferred direction.

It is worth noting that the joint distribution focuses solely on the cosθ parameter, as the azimuthal angle ϕ exhibits isotropy by symmetry of the system. This characteristic becomes evident in Fig. 5 (lower right panel), which shows the ϕ – θ distribution of the source in the subsequent stage of the approach where we generate the electron source for Geant4-DNA water radiolysis simulations. This panel also reaffirms the earlier observation that most electrons propagate in the initial beam direction. However, it is essential to acknowledge that some electrons deviate from this path. The beam traverses a layer of water before reaching the sample, which itself is nearly 1 cm thick and thus subjected to numerous physical interactions.

The spatial distribution of the source provides another layer of insight into the main direction of electron propagation (Fig. 5). This distribution effectively complements the joint distribution of electron energy and direction showcased in Fig. 4. It underscores the dominant trend whereby electrons primarily propagate in the initial beam direction, that is along the z-axis of the Cartesian coordinate system. This consistency across multiple representations confirms the coherence of our modeling and simulation efforts in capturing the interplay between electron energy, direction, and spatial distribution within the irradiation process.

Regarding the spectral distribution, Fig. 4 also displays the electron energy distribution alone, providing a detailed view of how energy is distributed within the irradiated sample. Notably, the electrons have undergone an energy loss of approximately 3 MeV relative to the primary energy of the beam. This loss is in line with the stopping power of $\:\sim$ 2 MeV/cm for electrons of this energy range^40^. This spectral distribution is presented in units of (µm^-2^ Gy^-1^), signifying a fluence value normalized by the dose deposited within the sample. Through a straightforward analytical calculation, we can retrieve the sum of all the energy bin contents of this spectral distribution, in other words, the total fluence. Consider a practical example involving an irradiation of 10 Gy. For electron beams with minimal ionizing energy, such as the eRT6, the mass stopping power equals $\:\sim$ 2 MeV cm^2^/g^40^. Dividing the delivered dose by this mass stopping power leads to an estimate of the fluence for this given dose, which approximates to 312 μm^-2^. This result is consistent with the sum of the bin contents, totaling 32 μm^-2^ Gy^-1^. This agreement underscores the accuracy of our simulation in representing the electrons within the sample.

### Investigated irradiation scenarios and quantification of radiolytic species production

Increasing the dose-per-pulse from 0.17 Gy to 10 Gy induces important changes in the temporal evolution of certain radio-induced species, as depicted in Fig. 6. The fall of hydroxyl radicals ($^{ \bullet } \text{O}\text{H}$) occurs at an earlier stage as the dose-per-pulse rises; higher dose-per-pulse results in a reduced production and lifetime of superoxide (${\text{O}}_{2}^{{\bullet\:}-}$); the amount of hydrogen peroxide (${\text{H}}_{2}{\text{O}}_{2}$) depends on the observation time point, with higher ${\text{H}}_{2}{\text{O}}_{2}$ shortly after pulse delivery for increased dose-per-pulse levels, but lower ${\text{H}}_{2}{\text{O}}_{2}$ toward the end of the simulation. The other radio-induced species (${\text{e}}_{\text{a}\text{q}}^{-}$, $\:\text{H}^{ \bullet }$, and $\text{H}{\text{O}}_{2}^{\bullet\:}$) appear minimally influenced by dose-per-pulse variations. The presence of 1% oxygen within water contributes to scavenging solvated electrons (${\text{e}}_{\text{a}\text{q}}^{-}$) and hydrogen radicals ($\text{H}^{ \bullet }$), with these reactions occurring early and limiting the impact of dose-per-pulse adjustments. The computation given during irradiation is normalized with respect to the total deposited energy from the pulse, limiting conclusive insights into events during the initial 1.8 µs of the simulation. In this context, a detailed computation of the *G* value during progressive energy deposition warrants consideration for a more comprehensive representation.

As energy is deposited in water, $^{ \bullet } \text{O}\text{H}$ increases until their mutual reaction, resulting in the production of ${\text{H}}_{2}{\text{O}}_{2}$, reaches a threshold where $^{ \bullet } \text{O}\text{H}$ begins to fall (see chemical reaction 1a^41^). The transition from an increase to a decrease in $^{ \bullet } \text{O}\text{H}$ leads to a brief delay in the production of ${\text{H}}_{2}{\text{O}}_{2}$. Once irradiation ceases, $^{ \bullet } \text{O}\text{H}$ continues to decrease and eventually becomes completely depleted, notably leading to the production of ${\text{H}}_{2}{\text{O}}_{2}$ but also the depletion of $\:{\text{O}}_{2}^{{\bullet\:}-}$, as shown by chemical reaction 1b^41^. This initial phase of the temporal evolution of $^{ \bullet } \text{O}\text{H}$, ${\text{H}}_{2}{\text{O}}_{2}$, and $\:{\text{O}}_{2}^{{\bullet\:}-}$ is mainly governed by the set of reactions 1a and 1b.

1a $$^{ \bullet } {\text{OH}} + \;^{ \bullet } {\text{OH}} \to {\text{H}}_{2} {\text{O}}_{2} \;\;\;\;\;\;\;\;\;\;\;\;\;\;\;\;\;\;\;\;\;\;\;\;\;\;\;\;\;\;\;\;{\text{k}}_{{{\text{1a}}}} = \;5.50 \cdot 10^{9} \;{\text{M}}^{{ - 1}} {\text{s}}^{{ - 1}}$$

1b $$^{ \bullet } {\text{OH}} + {\text{O}}_{2}^{{\bullet\:}-} \to {\text{O}}_{2} + {\text{OH}}^{ - } \;\;\;\;\;\;\;\;\;\;\;\;\;\;\;\;\;\;\;\;\;\;\;\;\;\;\;{\text{k}}_{{{\text{1b}}}} = \;1.07 \cdot 10^{{10}} \;{\text{M}}^{{ - 1}} {\text{s}}^{{ - 1}}$$

The final increase in ${\text{H}}_{2}{\text{O}}_{2}$ is closely tied to the evolution of $\:{\text{O}}_{2}^{{\bullet\:}-}$, both of which are governed by the following set of chemical reactions (2a, 2b, 2c, and 2d^41^). Chemical reactions 2a and 2b contribute to the depletion of $\:{\text{O}}_{2}^{{\bullet\:}-}$ and the formation of $\text{H}{\text{O}}_{2}^{-}$, which is immediately converted into ${\text{H}}_{2}{\text{O}}_{2}$ through reactions 2c and 2d. Either of these two chemical reactions alone is sufficient for ${\text{H}}_{2}{\text{O}}_{2}$ production.

2a $${\text{H}}_{3} {\text{O}}^{ + } + {\text{O}}_{2}^{{\bullet\:}-} \to {\text{HO}}_{2}^{\bullet\:} + {\text{H}}_{2} {\text{O}}\;\;\;\;\;\;\;\;\;\;\;\;\;\;\;\;\;\;\;\;\;\;\;{\text{k}}_{{{\text{2a}}}} = \;4.78 \cdot 10^{{10}} \;{\text{M}}^{{ - 1}} {\text{s}}^{{ - 1}}$$

2b $${\text{HO}}_{2}^{\bullet\:} + {\text{O}}_{2}^{{\bullet\:}-} \to {\text{HO}}_{2}^{ - } + {\text{O}}_{2} \;\;\;\;\;\;\;\;\;\;\;\;\;\;\;\;\;\;\;\;\;\;\;\;\;\;\;{\text{k}}_{{{\text{2b}}}} = \;9.70 \cdot 10^{7} \;{\text{M}}^{{ - 1}} {\text{s}}^{{ - 1}}$$

2c $${\text{H}}_{3} {\text{O}}^{ + } + {\text{HO}}_{2}^{ - } \to {\text{H}}_{2} {\text{O}}_{2} + {\text{H}}_{2} {\text{O}}\;\;\;\;\;\;\;\;\;\;\;\;\;\;\;\;\;\;\;\;{\text{k}}_{{{\text{2c}}}} = \;5.00 \cdot 10^{{10}} \;{\text{M}}^{{ - 1}} {\text{s}}^{{ - 1}}$$

2d $${\text{HO}}_{2}^{ - } + {\text{H}}_{2} {\text{O}} \to {\text{H}}_{2} {\text{O}}_{2} + {\text{OH}}^{ - } \;\;\;\;\;\;\;\;\;\;\;\;\;\;\;\;\;\;\;\;\;{\text{k}}_{{{\text{2d}}}} = \;1.36 \cdot 10^{{6}} \;{\text{M}}^{{ - 1}} {\text{s}}^{{ - 1}}$$

One of the critical free-radical species induced by ionizing radiation is $^{ \bullet } \text{O}\text{H}$, which actively attacks molecules in close proximity to its production site, leading to cellular damage^42^. These highly reactive species can cause substantial harm to cellular components, including DNA, proteins, and lipids, especially under stress conditions like ionizing radiation exposure (oxidative stress). This damage can result in various adverse effects, such as DNA strand breaks, which can trigger mutations, cell death, and contribute to the development of radio-induced diseases, including cancer. The importance of reactive oxygen species (ROS) such as $\:{\text{O}}_{2}^{{\bullet\:}-}$ and ${\text{H}}_{2}{\text{O}}_{2}$ in terms of cellular damage primarily lies in their ability to potentially generate $^{ \bullet } \text{O}\text{H}$^43^. As shown in Fig. 6, $^{ \bullet } \text{O}\text{H}$ has a shorter lifetime, and less $\:{\text{O}}_{2}^{{\bullet\:}-}$ are generated at higher dose-per-pulse levels. On the other hand, the amount of ${\text{H}}_{2}{\text{O}}_{2}$ tends to be higher with increased dose-per-pulse after pulse delivery, although it is lower toward the end of the simulation. This study focused on the production of radio-induced species within pure water with 1% oxygen. The cellular environment is far more complex, and future investigations should consider additional components and processes to provide a more comprehensive understanding of the impact of ultra-high dose rate irradiation on biological tissue, and its potential implications in the FLASH effect.

The rate and quantity of $$^{ \bullet } \text{O}\text{H}$$ production from ROS depend on cellular antioxidants and available reducing systems for $${\text{H}}_{2}{\text{O}}_{2}$$. $$^{ \bullet } \text{O}\text{H}$$ can arise from $${\text{H}}_{2}{\text{O}}_{2}$$ in the presence of reduced transition metals such as Fe^2+^. If appropriate reducing species are available to convert Fe^3+^ to Fe^2+^, $$^{ \bullet } \text{O}\text{H}$$ production from $${\text{H}}_{2}{\text{O}}_{2}$$ can proceed cyclically. $$\:{\text{O}}_{2}^{{\bullet\:}-}$$ is one of the potential reducing agents involved in this process (Haber-Weiss reaction)^43^. This interplay of radio-induced species within the cellular environment highlights the complexity of processes that deserve further investigation under the irradiation conditions presented in this study.

Concerning the pulse temporal structure, Fig. 7 shows evidence that this factor does not influence the long-term evolution of species. Simulating the details of the pulse structure may not be necessary as demonstrated by the modeled ultra-high dose rate irradiation representing a typical irradiation from the eRT6 electron linac. This observation considerably simplifies the simulation procedure when investigating the evolution of species beyond the pulse duration. However, it is essential to exercise caution and evaluate the real-time *G* value during irradiation if the critical time scale for mechanisms related to the FLASH effect occurs around these early time points.

## Conclusion

The objective of this study was to simulate water radiolysis under ultra-high dose rate conditions using the FLASH-validated Oriatron eRT6 linac as an electron source. We explored the impact of dose-per-pulse on the production of radio-induced species in water and the role of pulse temporal structure in modeling ultra-high dose rate irradiation. The Geant4-based design of the eRT6 linac proved effective, aligning well with experimental data. Verification of the electron source generated from this eRT6 model and adjusted for Geant4-DNA simulations confirmed our efforts to capture the correlation between electron energy, direction, and spatial distribution within the irradiated sample. This model allowed the observation of important changes in the temporal evolution of certain species, such as the earlier fall in hydroxyl radicals ($^{ \bullet } \text{O}\text{H}$) and reduced production and lifetime of superoxide (${\text{O}}_{2}^{{\bullet\:}-}$) with higher dose-per-pulse. The pulse temporal structure did not influence the long-term evolution of species.

While this study focused on pure water with 1% oxygen, the cellular environment is much more complex. In the context of the FLASH effect, future research should consider additional components and processes to better understand the impact of ultra-high dose rate irradiation on biological tissue. Our findings emphasize the need for further investigations into the interplay of radio-induced species with the cellular environment under these conditions, including irradiation scenarios different from those presented here, such as multi-pulse irradiation encountered in FLASH effect studies. Other particle types – protons, heavy ions – should also be investigated in future studies.

## Electronic supplementary material

Below is the link to the electronic supplementary material.

Supplementary Material 1

## Data Availability

The databases used and analyzed in this study are available from the corresponding author upon reasonable request.
