# Supplementary material for: Investigating ultra-high dose rate water radiolysis using the Geant4-DNA toolkit and a Geant4 model of the Oriatron eRT6 electron linac
Source: Sci Rep. 2024 Nov 4;14:26707. doi: 10.1038/s41598-024-76769-0 (PMC11535405; doi:10.1038/s41598-024-76769-0)
Supplement: Supplementary file 1 — Supplementary Material 1 [file 41598_2024_76769_MOESM1_ESM.docx]

**Supporting Materials**

**Table S1.** List of chemical reactions used to simulate water radiolysis at ultra-high dose rate.

| Chemical reaction | Reaction rate (M^-1^ s^-1^) [1] | Type – for IRT method [1] |
| --- | --- | --- |
| $H$^•^ + $e_{\mathrm{aq}}^{-}$ + $H_{2}O$ → $H_{2}$ + $\mathrm{OH}^{-}$ | 2.50 x 10^10^ | 1 |
| $H$^•^ + $H$^•^ → $H_{2}$ | 5.03 x 10^9^ | 1 |
| $H$^•^ + $O^{\bullet-}$ → $\mathrm{OH}^{-}$ | 2.00 x 10^10^ | 1 |
| $O$(^3^$P$) + ^•^$\mathrm{OH}$ → $HO_{2}^{\bullet}$ | 2.02 x 10^10^ | 1 |
| $O$(^3^$P$) + $H$^•^ → ^•^$\mathrm{OH}$ | 2.02 x 10^10^ | 1 |
| $O$(^3^$P$) + $HO_{2}^{\bullet}$ → $O_{2}$ + ^•^$\mathrm{OH}$ | 2.02 x 10^10^ | 1 |
| $O$(^3^$P$) + $O$(^3^$P$) → $O_{2}$ | 2.20 x 10^10^ | 1 |
| ^•^$\mathrm{OH}$ + $e_{\mathrm{aq}}^{-}$ → $\mathrm{OH}^{-}$ | 2.95 x 10^10^ | 2 |
| ^•^$\mathrm{OH}$ + ^•^$\mathrm{OH}$ → $H_{2}O_{2}$ | 5.50 x 10^9^ | 2 |
| ^•^$\mathrm{OH}$ + $\mathrm{OH}^{-}$ → $O^{\bullet-}$ + $H_{2}O$ | 1.18 x 10^10^ | 2/6 |
| ^•^$\mathrm{OH}$ + $\mathrm{HO}_{2}^{-}$ → $HO_{2}^{\bullet}$ + $\mathrm{OH}^{-}$ | 8.32 x 10^9^ | 2 |
| ^•^$\mathrm{OH}$ + $O^{\bullet-}$ → $\mathrm{HO}_{2}^{-}$ | 1.00 x 10^9^ | 2 |
| ^•^$\mathrm{OH}$ + $HO_{2}^{\bullet}$ → $O_{2}$ + $H_{2}O$ | 7.90 x 10^9^ | 2 |
| ^•^$\mathrm{OH}$ + $H_{2}O_{2}$ → $HO_{2}^{\bullet}$ + $H_{2}O$ | 2.88 x 10^7^ | 2 |
| ^•^$\mathrm{OH}$ + $H_{2}$ → $H$^•^ + $H_{2}O$ | 3.28 x 10^7^ | 2 |
| ^•^$\mathrm{OH}$ + $O_{2}^{\bullet-}$ → $O_{2}$ + $\mathrm{OH}^{-}$ | 1.07 x 10^10^ | 2 |
| ^•^$\mathrm{OH}$ + $O_{3}^{\bullet-}$ → $O_{2}^{\bullet-}$ + $HO_{2}^{\bullet}$ | 8.50 x 10^9^ | 2 |
| $H$^•^ + ^•^$\mathrm{OH}$ → $H_{2}O$ | 1.55 x 10^10^ | 2 |
| $H$^•^ + $\mathrm{OH}^{-}$ → $e_{\mathrm{aq}}^{-}$ + $H_{2}O$ | 2.51 x 10^7^ | 2/6 |
| $H$^•^ + $HO_{2}^{\bullet}$ → $H_{2}O_{2}$ | 1.00 x 10^10^ | 2 |
| $H$^•^ + $H_{2}O_{2}$ → ^•^$\mathrm{OH}$ + $H_{2}O$ | 3.50 x 10^7^ | 2 |
| $H$^•^ + $O_{2}^{\bullet-}$ → $\mathrm{HO}_{2}^{-}$ | 1.00 x 10^10^ | 2 |
| $e_{\mathrm{aq}}^{-}$ + $H_{2}O_{2}$ → $\mathrm{OH}^{-}$ + ^•^$\mathrm{OH}$ | 1.10 x 10^10^ | 2 |
| $e_{\mathrm{aq}}^{-}$ + $HO_{2}^{\bullet}$ → $\mathrm{HO}_{2}^{-}$ | 1.29 x 10^10^ | 2 |
| $H_{2}O_{2}$ + $\mathrm{OH}^{-}$ → $\mathrm{HO}_{2}^{-}$ + $H_{2}O$ | 1.18 x 10^10^ | 2/6 |
| $H_{2}O_{2}$ + $O^{\bullet-}$ → $HO_{2}^{\bullet}$ + $\mathrm{OH}^{-}$ | 5.55 x 10^8^ | 2 |
| $O^{\bullet-}$ + $H_{2}$ → $H$^•^ + $\mathrm{OH}^{-}$ | 1.21 x 10^8^ | 2 |
| $HO_{2}^{\bullet}$ + $\mathrm{OH}^{-}$ → $O_{2}^{\bullet-}$ + $H_{2}O$ | 6.30 x 10^9^ | 2/6 |
| $HO_{2}^{\bullet}$ + $HO_{2}^{\bullet}$ → $H_{2}O_{2}$ + $O_{2}$ | 9.80 x 10^5^ | 2 |
| $HO_{2}^{\bullet}$ + $O_{2}^{\bullet-}$ → $\mathrm{HO}_{2}^{-}$ + $O_{2}$ | 9.70 x 10^7^ | 2 |
| $O$(^3^$P$) + $H_{2}O_{2}$ → $HO_{2}^{\bullet}$ + ^•^$\mathrm{OH}$ | 1.60 x 10^9^ | 2 |
| $O$(^3^$P$) + $H_{2}$ → $H$^•^ + ^•^$\mathrm{OH}$ | 4.77 x 10^3^ | 2 |
| $O$(^3^$P$) + $\mathrm{OH}^{-}$ → $\mathrm{HO}_{2}^{-}$ | 4.20 x 10^8^ | 2/6 |
| $O$(^3^$P$) + $\mathrm{HO}_{2}^{-}$ → $O_{2}^{\bullet-}$ + ^•^$\mathrm{OH}$ | 5.30 x 10^9^ | 2 |
| $e_{\mathrm{aq}}^{-}$ + $e_{\mathrm{aq}}^{-}$ + 2 $H_{2}O$ → $H_{2}$ + 2 $\mathrm{OH}^{-}$ | 6.36 x 10^9^ | 3 |
| $H_{3}O^{+}$ + $\mathrm{OH}^{-}$ → 2 $H_{2}O$ | 1.13 x 10^11^ | 3/6 |
| $H_{3}O^{+}$ + $O_{3}^{\bullet-}$ → ^•^$\mathrm{OH}$ + $O_{2}$ + $H_{2}O$ | 9.00 x 10^10^ | 3/6 |
| $e_{\mathrm{aq}}^{-}$ + $H_{3}O^{+}$ → $H$^•^ + $H_{2}O$ | 2.11 x 10^10^ | 4/6 |
| $e_{\mathrm{aq}}^{-}$ + $O^{\bullet-}$ → 2 $\mathrm{OH}^{-}$ | 2.31 x 10^10^ | 4 |
| $e_{\mathrm{aq}}^{-}$ + $O_{2}^{\bullet-}$ + 2 $H_{2}O$ → $H_{2}O_{2}$+ 2 $\mathrm{OH}^{-}$ | 1.29 x 10^10^ | 4 |
| $e_{\mathrm{aq}}^{-}$ + $\mathrm{HO}_{2}^{-}$ → $O^{\bullet-}$ + $\mathrm{OH}^{-}$ | 3.51 x 10^9^ | 4 |
| $H_{3}O^{+}$ + $\mathrm{HO}_{2}^{-}$ → $H_{2}O_{2}$ + $H_{2}O$ | 5.00 x 10^10^ | 4/6 |
| $H_{3}O^{+}$ + $O^{\bullet-}$ → ^•^$\mathrm{OH}$ + $H_{2}O$ | 4.78 x 10^10^ | 4/6 |
| $H_{3}O^{+}$ + $O_{2}^{\bullet-}$ → $HO_{2}^{\bullet}$ + $H_{2}O$ | 4.78 x 10^10^ | 4/6 |
| $O^{\bullet-}$ + $O^{\bullet-}$ → $H_{2}O_{2}$ + 2 $\mathrm{OH}^{-}$ | 1.00 x 10^8^ | 4 |
| $O^{\bullet-}$ + $O_{3}^{\bullet-}$ → 2 $O_{2}^{\bullet-}$ | 7.00 x 10^8^ | 4 |
| $O^{\bullet-}$ + $O_{2}^{\bullet-}$ + $H_{2}O$ → $O_{2}$ + 2 $\mathrm{OH}^{-}$ | 6.00 x 10^8^ | 4 |
| $O^{\bullet-}$ + $\mathrm{HO}_{2}^{-}$ → $O_{2}^{\bullet-}$ + $\mathrm{OH}^{-}$ | 3.50 x 10^8^ | 4 |
| $O_{2}^{\bullet-}$ + $O_{2}^{\bullet-}$ + 2 $H_{2}O$ → $H_{2}O_{2}$ + $O_{2}$ + 2 $\mathrm{OH}^{-}$ | 1.00 x 10^2^ | 4 |
| $O_{2}$ + $e_{\mathrm{aq}}^{-}$ → $O_{2}^{\bullet-}$ | 1.74 x 10^10^ | 6 |
| $O_{2}$ + $H$^•^ → $HO_{2}^{\bullet}$ | 2.10 x 10^10^ | 6 |
| $O_{2}$ + $O^{\bullet-}$ → $O_{3}^{\bullet-}$ | 3.70 x 10^9^ | 6 |
| $O_{2}$ + $O$(^3^$P$) → $O_{3}$ | 4.00 x 10^9^ | 6 |
| $H$^•^ + $H_{2}O$ → $H_{3}O^{+}$ + $e_{\mathrm{aq}}^{-}$ | 1.07 x 10^-1^ | 6 |
| ^•^$\mathrm{OH}$ + $H_{2}O$ → $H_{3}O^{+}$ + $O^{\bullet-}$ | 1.17 x 10^-3^ | 6 |
| $HO_{2}^{\bullet}$ + $H_{2}O$ → $H_{3}O^{+}$ + $O_{2}^{\bullet-}$ | 1.20 x 10^4^ | 6 |
| $H_{2}O_{2}$ + $H_{2}O$ → $H_{3}O^{+}$ + $\mathrm{HO}_{2}^{-}$ | 1.17 x 10^-3^ | 6 |
| $O^{\bullet-}$ + $H_{2}O$ → $\mathrm{OH}^{-}$ + ^•^$\mathrm{OH}$ | 1.36 x 10^6^ | 6 |
| $e_{\mathrm{aq}}^{-}$ + $H_{2}O$ → $\mathrm{OH}^{-}$ + $H$^•^ | 1.58 x 10^1^ | 6 |
| $\mathrm{HO}_{2}^{-}$ + $H_{2}O$ → $\mathrm{OH}^{-}$ + $H_{2}O_{2}$ | 1.36 x 10^6^ | 6 |
| $O_{2}^{\bullet-}$ + $H_{2}O$ → $\mathrm{OH}^{-}$ + $HO_{2}^{\bullet}$ | 1.50 x 10^-1^ | 6 |
| $O$(^3^$P$) + $H_{2}O$ → ^•^$\mathrm{OH}$ + ^•^$\mathrm{OH}$ | 1.90 x 10^3^ | 6 |
| $O_{3}^{\bullet-}$ → $O^{\bullet-}$ + $O_{2}$ | 2.66 x 10^3^ s^-1^ | 6 |

**References**

[1] Plante I, Devroye L. Considerations for the independent reaction times and step-by-step methods for radiation chemistry simulations. Radiat Phys Chem 2017;139:157-172. https://doi.org/10.1016/j.radphyschem.2017.03.021
